# 3T MRI induced post-traumatic stress disorder: a case report

**DOI:** 10.1186/1755-7682-5-27

**Published:** 2012-10-10

**Authors:** Shaheen E Lakhan

**Affiliations:** 1 Neurological Institute, Cleveland Clinic, 9500 Euclid Ave, S100A, Cleveland, OH, 44106, USA

**Keywords:** PTSD, MRI, Imaging, Anxiety, Stress

## Abstract

**Introduction:**

MRI is considered a safe and well tolerated imaging technique with risks largely limited to heating and/or displacement of implanted ferromagnetic metal in the patient’s body, worsening anxiety, triggering claustrophobia, and gadolinium induced nephrogenic systemic fibrosis.

**Case presentation:**

We present a case of a 26 year old Asian American man with no significant past medical or psychiatric history and two months of left T4 radicular pain. During 3T-MRI of the whole spine, the patient experienced acute agitation, fear, anxiety, tachypnea, tachycardia with palpitations, and dizziness. He felt intense surface heat over segments of his body and very loud noises. He perceived impending serious bodily harm by the scanner. The scan was aborted at the lumbar spine, and cervical and thoracic spine was unremarkable. The patient’s pain resolved in the weeks following with over the counter analgesics, however, he developed increased arousal, re-experiencing the event, persistent avoidance, and significant psychosocial impairment consistent with DSM-IV-TR criteria for post-traumatic stress disorder (PTSD).

**Conclusion:**

This is the first reported case of MRI induced PTSD. Theoretically, the high-magnetic field of the 3T scanner may have contributed to the development of symptoms.

## Introduction

MRI is generally considered a safe and well tolerated imaging technique that has advanced the field of medicine by leaps and bounds. Risks to the patient are largely derived from the magnetic field and contrast agent (gadolinium) and range from heating tattoos to nephrogenic systemic fibrosis. Also, the noise, confined environment especially in closed scanners, and tissue heating properties can worsen anxiety and trigger claustrophobia.

Here, we describe a case of post-traumatic stress disorder (PTSD) following a “traumatic” 3T-MRI scan in a large tertiary medical center. The DSM-IV-TR diagnosis of PTSD involves experiencing a traumatic event, persistently re-experiencing the event, persistent avoidance, persistent symptoms of more than one month’s duration, and significant psychosocial/occupational impairment [1].

## Case presentation

A 26 year old Asian American man with no significant past medical history presented to his primary care provider for mid back pain of three months duration. The pain was sharp, 5/10 in intensity, intermittent, and left sided radicular in nature localizing to the T5 dermatome and partially-relieved by NSAIDs. The physical and neurological exams were unremarkable. The patient had no HIV, Lyme, or tuberculosis risk factors, and denies recent illnesses or infections. He was asked to continue to take NSAIDs for the pain and undergo MRI of the cervical, thoracic, and lumbar spine without contrast to rule out structural disease and look for evidence of radiculopathy.

The patient underwent 3T MRI at a tertiary medical center in a traditional closed scanner. The patient was provided with ear plugs. During the lumbar portion of the scan, the patient became acutely agitated, felt extremely hot, tachypneic, tachycardic with palpitations, intense anxiety and feeling of impending doom, and acute vertigo. He heard very loud noises and areas of intense heat over his external body. He thought he would sustain serious injury to his body by the MRI scanner. The scan was aborted and the patient was unsteady on his feet momentarily afterwards. He appeared frightened. As the patient had no history of claustrophobia or anxiety disorder, he was not premedicated with benzodiazepines. The cervical and thoracic spine MRI were unremarkable, and lumbar spine incomplete and no interpretation could be made.

Over the next two to four weeks, the mid back pain resolved. However, over the next three months, the patient developed increased arousal (difficulty sleeping and hypervigilance) and flashbacks of the MRI encounter with recurrence of symptoms (fear, tachypneic, tachycardia with palpitations, dizziness, and anxiety). He would feel as if the traumatic event was recurring. Moreover, he avoided further MRI scans, loud repetitive noises as experienced in the MR scanner, and reminders of the scan. He experienced difficulty falling asleep and concentrating.

He presented three months after the MRI. The structural clinical interview for DSM-IV (SCID) confirmed the diagnosis of PTSD. Specifically, the patient’s constellation of symptoms, duration (longer than one month), and impairment of psychosocial functioning met PTSD criteria. The top differential diagnoses were panic attack and panic disorder. Careful history taking did not reveal any past evidence of panic attack or other anxiety disorders. There were no other significant traumatic events or abuse. Although PTSD can result from panic, it is often less severe than prototypical external traumas such as rape or combat [2]. The temporal association of the external traumatic event with onset of hyperarousal, intrusions, and avoidance symptoms favors PTSD.

The patient refused pharmacological therapy and no further workup for his transient radicular pain was initiated.

## Discussion

This is the first published report of PTSD following “traumatic” MRI. The patient in our case had no prior traumatic event or psychiatric history. Patients with anxiety and claustrophobia are often premedicated with benzodiazepines, however, absent this history, our patient was not.

In this case, a 3T MRI was used which has a stronger magnetic field than a 1.5T scanner -- the current clinical benchmark. The 3T has twice the noise levels of the 1.5T and may exceed 130dBA [3]. Likewise, 3T has quadruple the energy deposited by a radiofrequency field in a given mass of tissue (specific absorption rate) than 1.5T which may lead to greater tissue heating [4]. These two factors may have contributed to the patients’ development of PTSD.

## Conclusion

Further data collection and research into the development of PTSD after high-magnetic field MRI is warranted.

## Consent

Written informed consent was obtained from the patient for publication of this case report and any accompanying images. A copy of the written consent is available for review by the Editor-in-Chief of this journal.

## Competing interests

SEL serves as an associate editor of the *Journal of Medical Case Reports* and *BMC Research Notes*, and board member of BioMed Central’s *International Archives of Medicine*.

## Author’s contributions

SEL acquired and interpreted the data, drafted the manuscript, and approved the version to be published.
